# Association between grip strength and hand and knee radiographic osteoarthritis in Korean adults: Data from the Dong-gu study

**DOI:** 10.1371/journal.pone.0185343

**Published:** 2017-11-30

**Authors:** Lihui Wen, Min-Ho Shin, Ji-Hyoun Kang, Yi-Rang Yim, Ji-Eun Kim, Jeong-Won Lee, Kyung-Eun Lee, Dong-Jin Park, Tae-Jong Kim, Sun-Seog Kweon, Young-Hoon Lee, Yong-Woon Yun, Shin-Seok Lee

**Affiliations:** 1 Department of Rheumatology, Chonnam National University Medical School & Hospital, Gwangju, Republic of Korea; 2 Department of Biomedical Sciences, Chonnam National University Medical School, Gwangju, Republic of Korea; 3 Department of Preventive Medicine, Chonnam National University Medical School, Gwangju, Republic of Korea; 4 Jeonnam Regional Cancer Center, Chonnam National University Hwasun Hospital, Hwasun, Republic of Korea; 5 Department of Preventive Medicine & Institute of Wonkwang Medical Science, Wonkwang University College of Medicine, Iksan, Republic of Korea; 6 Department of Preventive Medicine, Bitgoeul Chonnam National University Hospital, Gwangju, Republic of Korea; National Cancer Center, JAPAN

## Abstract

**Objectives:**

We assessed whether grip strength was related to various types of radiographic damage in Korean adults with osteoarthritis (OA).

**Methods:**

Data from 2,251 subjects enrolled in the Dong-gu study, who had no hand joint pain, were analyzed to investigate the relationship between grip strength and OA. Hand grip strength was measured using a hand-held dynamometer, and radiographs of the hand and knee were scored according to a semi-quantitative grading system. Multiple linear regressions were used to explore associations between grip strength and radiographic features of OA.

**Results:**

Grip strength in men and women was negatively related to hand (both *p* < 0.001) and knee (men, *p* < 0.001; women, *p* = 0.010) OA after adjusting for confounders. Hand (men, *p* < 0.001; women, *p* = 0.001) and knee (both *p* < 0.001) joint space narrowing (JSN) showed the strongest associations with low grip strength, regardless of gender. Moreover, the severity of hand osteophytes in women (*p* = 0.001), and subchondral cysts (men, *p* < 0.001) was correlated with low grip strength in both genders.

**Conclusions:**

Among subjects without hand joint pain, low grip strength was associated significantly with hand and knee radiographic OA, regardless of gender. Among all types of OA radiographic damage, low grip strength showed the strongest association with JSN.

## Introduction

Osteoarthritis (OA) is the most common joint disease in older adults; its main symptoms are pain and joint deformation. OA frequently leads to physical disability and social limitations [1]. The hand is often involved in cases of OA, although disability related specifically to hand OA has received little attention. In particular, grip strength is a major determinant of hand disability [2].

Recently, several cross-sectional studies showed that hand OA had a significant negative relationship with grip strength [3–9]. For instance, Lee *et al*. [4] revealed that the sums of Kellgren and Lawrence grades for thumbs and middle fingers were negatively associated with grip strength. Additionally, Ding *et al*. [5] indicated that symptomatic hand OA increased the risk of low pinch grip strength in both hands. Moreover, in a study by Dominick *et al*. [6], increasing radiographic severity of hand OA was associated with reduced grip and pinch strength, even after controlling for self-reported pain. Similarly, another study [7] showed that grip strength was lower in hand OA patients with distal interphalangeal and proximal interphalangeal joint involvement. In contrast, Chaisson et al. found that subjects with high maximal grip strength were at increased risk for the development of hand OA in a longitudinal study [10]. The difference in results between cross-sectional study and longitudinal study may have been due to the presence of accompanying hand joint pain in some study subjects; previous studies suggested that hand function was associated with pain and tenderness, rather than with the radiological grade of hand OA [11], and that poor physical function was due to increased pain, which was indirectly associated with increasing severity of radiographic hand OA leading to worse bone mineral density results [12], Thus, assessment of the relationship between grip strength and hand OA in the absence of pain is important, as it would demonstrate a one-way effect of hand OA on grip strength.

Although some studies have shown a negative relationship between grip strength and hand OA, little is known about how grip strength is related to specific radiographic features of hand OA. In previous studies, OA was typically defined using the Kellgren-Lawrence (K-L) grading system, which reflects only gross clinical severity and overall severity grades. Thus, the use of a more detailed quantitative grading system to evaluate the radiographic features of OA is necessary [13]; such a system can show more clearly how grip strength is related to detailed radiographic features of OA.

Several studies have analyzed the relationship between grip strength and hand OA. However, no reported study has examined whether grip strength, as a measure of muscle activity, is related to knee OA. Although knee OA has been shown to be induced by weight-bearing activities, as a result of excessive mechanical forces, it remains unclear whether muscle strength is a risk factor for knee OA. Through this study, we aim to analyze whether knee OA is affected by weight alone, or complicated by limb-muscle strength. Theoretically, this study may show a one-way effect of reduced muscle strength on knee OA.

In this large, population-based cohort study, we took advantage of the availability of subjects without hand pain to evaluate the effect of grip strength on OA using a novel, semi-quantitative grading system. We also examined whether grip strength was related to detailed radiographic features of OA.

## Methods

### Study design and population

This research formed part of the Dong-gu study, which is an ongoing prospective study designed to investigate the prevalence, incidence, and risk factors for chronic disease among 9,260 subjects aged ≥ 50 years, as described previously [14]. Baseline data were collected from 2007 to 2010 in Dong-gu, Gwangju City, Republic of Korea. X-rays of knees and hands were obtained from 2,489 out of 2,516 participants in the 2009 baseline examination. Of these, 51 subjects with a past history of knee replacement surgery or knee amputation, and 23 subjects with missing data on grip strength, were excluded. We further excluded 164 subjects with hand joint pain to ensure that the association between grip strength and OA would not be affected by pain in the hand. Finally, the remaining 2,251 subjects were used in the analysis. All participants provided written informed consent at the time of enrolment into the study, and the study was approved by the Institutional Review Board of Chonnam National University Hospital (IRB No. CNUH-2015-123).

### Covariates

Smoking status, alcohol consumption, and education were assessed using a standardized questionnaire. Smoking status was classified as non-smoker, including both never smoker (< 100 lifetime cigarettes and not currently smoking) and former smoker (> 100 lifetime cigarettes and not currently smoking), and current smoker (> 100 lifetime cigarettes and currently a smoker). Alcohol consumption (within the past 12 months) was defined as current drinker or non-drinker. Education was categorized as middle school or less and high school or more. Height and weight were measured to calculate body mass index (BMI). Hand joint pain was defined by responses to a questionnaire; participants were asked whether they had experienced any hand joint pain in the past month and, if yes, the number of days on which this had occurred. Hand joint pain was classified as positive if a subject responded “yes” to the joint pain question and indicated that the pain had lasted for “more than 0 days”.

### Grip strength

Hand grip strength was measured using a hand-held dynamometer (SH-2000D; O2RUN Co., Ltd., Seoul, Korea). Subjects held the dynamometer while seated, with the shoulder adducted and rotated neutrally and the elbow flexed at 90°. They were instructed to test the right hand and left hand twice each, unless they were unable to perform the test with one or both hands because of a history of a specific injury, such as a previously broken wrist or stroke; such cases were excluded from the analysis. We used the maximum value from among the four measures of grip strength in the final analysis.

### Assessment of radiographic features

X-rays of both knees and hands were obtained using a computed radiography X-ray system with the participants in a standing position. All images were anteroposterior radiographs depicting both knees or hands on a single X-ray film. Radiographs were scored according to a semi-quantitative grading method, using the Atlas of Standard Radiographs, by two trained observers who were blinded to the clinical data. Then, based on this semi-quantitative grading method [13], the total OA score was obtained by summing the scores for each radiographic feature (see below). The initial results from the two observers were compared, and the radiographs were reviewed by a third independent observer in cases of disagreement. Interobserver and intraobserver reliability were tested using a subgroup of participants from the Dong-gu study. The evaluation was performed twice on 100 randomly chosen radiographs (50 radiographs of the knee, 50 radiographs of the hand) by the same observers 1 month apart. Kappa statistics were used to ascertain interobserver (*κ* = 0.79–0.89) and intraobserver (*κ* = 0.85–0.92) reproducibility values.

Individual radiographic features were recorded for the hand (distal interphalangeal joint [DIP], proximal interphalangeal joint [PIP], trapeziometacarpal joint [CMC], interphalangeal joint of the thumb [IP], and naviculotrapezial joint [NTJ]), and knee (medial compartment, lateral compartment, tibial component, and femoral component). The extent of primary osteophytes and joint space narrowing (JSN) at primary sites (DIP, PIP, CMC, IP, NTJ, medial and lateral compartments for knee JSN, and medial femoral condyle, medial tibial plateau, lateral femoral condyle, and lateral tibial plateau for knee osteophytes) were graded from 0 to 3 (i.e., 0 = normal, 1 = mild change, 2 = moderate change, 3 = severe change). Other abnormalities (malalignment, erosion, subchondral sclerosis, subchondral cysts, and attrition) and OA at other sites (IP and NTJ) were graded as absent (0) or present (1). Using this semi-quantitative grading system, total OA scores were computed by summing the scores of individual radiographic features.

The semi-quantitative grading system yielded total OA scores (max. 42) for the knee. The components thereof, including osteophyte (max. 24), JSN (max. 12), tibial attrition (max. 4), and sclerosis (max. 2) scores, were calculated and listed as means ± SDs. For the hand, osteoarthritic total score (max. 70), and osteophyte (max. 22), JSN (max. 22), subchondral cyst (max. 4), sclerosis (max. 6), erosion (max. 10), and malalignment (max. 6) scores were calculated. Of these, the osteophyte and JSN scores comprised the major proportions of the total scores for the knee (85.7%) and hand (62.9%).

### Statistical analysis

Student’s *t*-tests or χ^2^ tests were used to compare means or proportions of baseline data according to gender. In the case of radiographic features with a range of more than 10 points such as total OA scores, the osteophyte scores and JSN scores, the relationship between grip strength and radiographic features of OA was assessed using linear regression analysis. For radiologic features with a range of less than 10 points such as tibial attrition, sclerosis, subchondral cyst and erosion scores, these variables were converted to dichotomous variables (no (0), yes (score >0)) and then logistic regression analysis was used to evaluate the relationship between grip strength and these variables.

In multivariate analysis, age, body mass index, smoking status, drinking status, and education level variables were adjusted. In the linear regression analysis, the regression coefficient, 95% confidence interval, and p value were presented. In addition, Partial eta-squared (%) and standard beta coefficients were calculated to evaluate the effect size of each radiographic feature in the model. In the logistic regression model, the odds ratio, 95% confidence interval, and p-value were calculated to evaluate the relationship between grip strength and radiologic features with restricted range. P values < 0.05 (two-tailed) and 95% confidence intervals that did not include the null point were taken to indicate statistical significance. All statistical analyses were performed using the SPSS for Windows software (ver. 20.0; SPSS Inc., Chicago, IL, USA).

## Results

### Baseline characteristics of the subjects stratified by gender

At the initial examination, 2,415 subjects were enrolled to test the relationships between grip strength and radiographic OA (S1 File). After exclusion of individuals with hand joint pain (*n* = 164), data from 2,251 subjects were analyzed. Baseline characteristics are shown in Table 1. The mean age was 64.0 (SD 8.2) years; 1,022 (45.4%) subjects were men and 1,229 (54.6%) subjects were women. The men were significantly older than the women (*p* < 0.001). Significant differences between men and women in other confounding factors, such as BMI, smoking, alcohol consumption, and education, were also found (all *p* < 0.001). The average BMI (24.7 ± 3.0 kg/m^2^) was higher in women; however, the proportions of smoking (1.8%), alcohol consumption (35.4%), and education level of high school or above (21.3%) were lower in women. According to the distribution of grip strength by age category (S1 Table), the mean grip strength decreased from 50 years to 80 years. Moreover, the mean grip strength of women was lower than that of men in every age category, and the overall average grip strength (22.0 ± 4.8 kg) was significantly lower in women.

**Table 1:** Baseline characteristics of subjects without hand joint pain stratified by gender.

	Total	Men	Women	*P* value
	*N* = 2,251	*n* = 1,022	*n* = 1,229	
Age, years	64.0 ± 8.2	64.9 ± 8.1	63.2 ± 8.2	<0.001
Body mass index, kg/m^2^	24.4 ± 2.9	24.0 ± 2.8	24.7 ± 3.0	<0.001
Smoking#, *n* (%)	285 (12.7)	263 (25.7)	22 (1.8)	<0.001
Alcohol consumption, *n* (%)	1,126 (50.0)	691 (67.6)	435 (35.4)	<0.001
Education*, *n* (%)	753 (33.5)	491 (48.0)	262 (21.3)	<0.001
Grip strength, kg	28.2 ± 8.9	35.7 ± 6.6	22.0 ± 4.8	<0.001
Hand				
Total score	16.7 ± 6.0	16.2 ± 5.6	17.1 ± 6.2	<0.001
Score of osteophyte	6.4 ± 2.1	6.9 ± 2.1	6.1 ± 2	<0.001
Score of JSN	8.5 ± 3.3	7.8 ± 3.0	9.0 ± 3.4	<0.001
Score of subchondral cyst	1.0 ± 1.2	0.6 ± 1.0	1.3 ± 1.3	<0.001
Score of sclerosis	0.4 ± 0.8	0.4 ± 0.9	0.3 ± 0.7	<0.001
Score of erosion	0.3 ± 0.9	0.2 ± 0.9	0.3 ± 0.9	0.236
Score of malalignment	0.1 ± 0.4	0.1 ± 0.5	0.1 ± 0.4	0.394
Knee				
Total score	14.3 ± 6.7	13.7 ± 5.8	14.9 ± 7.4	<0.001
Score of osteophyte	7.5 ± 3.6	7.2 ± 3	7.8 ± 4.1	<0.001
Score of JSN	6 ± 2.5	5.7 ± 2.4	6.2 ± 2.6	<0.001
Score of tibial attrition	0.2 ± 0.6	0.2 ± 0.5	0.2 ± 0.6	0.248
Score of sclerosis	0.6 ± 0.9	0.5 ± 0.8	0.6 ± 0.9	0.001

# Current smoker

* High school or more

Unless otherwise indicated, data are shown as means ± standard deviations

### Associations between grip strength and hand OA

After adjusting for confounding factors (age, BMI, smoking, alcohol consumption, education) that might affect the risk of OA, we evaluated the associations between grip strength and hand radiographic OA scores in a total of 2,251 subjects (Tables 2 and 3). After Bonferroni correction for multiple comparison, higher total scores for hand radiographic OA were shown to significantly lower grip strength in both genders (both p < 0.001). Similarly, other dependent variables, such as JSN in men and women (*p* < 0.001 and *p* = 0.001, respectively), subchondral cysts in men (*p* < 0.001) showed negative correlations with grip strength. However, osteophytes showed a significant negative association with grip strength only in women (*p* = 0.001). Among all dependent variables, the strongest associations were between grip strength and hand JSN in men (eta = 0.036) and between grip strength and total hand radiographic OA score in women (eta = 0.015). When we evaluated the associations between grip strength and hand radiographic OA scores in a total of 2,415 subjects, the same results were found regardless of hand joint pain (S2 Table).

**Table 2:** Coefficients from a linear regression model examining the association of grip strength (kg) with total and individual radiographic feature scores of hand and knee osteoarthritis.

		Men (*n* = 1,022)				Women (*n* = 1,229)			
		Beta (95% CI)	Standard Beta	Eta	*P* value	Beta (95% CI)	Standard Beta	Eta	*P* value
Hand	Total score	-0.146 (-0.199, -0.093)	-0.172	0.028	<0.001*	-0.137 (-0.200, -0.075)	-0.106	0.015	<0.001*
	Osteophyte	-0.018 (-0.040, 0.004)	-0.057	0.003	0.101	-0.041 (-0.065, -0.016)	-0.100	0.009	0.001*
	JSN	-0.089 (-0.117, -0.060)	-0.198	0.036	<0.001*	-0.061 (-0.097, -0.025)	-0.087	0.009	0.001*
Knee	Total score	-0.110 (-0.169, -0.051)	-0.126	0.013	<0.001*	-0.107 (-0.188, -0.025)	-0.070	0.005	0.010
	Osteophyte	-0.044 (-0.076, -0.012)	-0.096	0.007	0.006	-0.034 (-0.080, 0.013)	-0.040	0.002	0.154
	JSN	-0.059 (-0.083, -0.034)	-0.160	0.021	<0.001*	-0.063 (-0.092, -0.033)	-0.115	0.014	<0.001*

Values are regression coefficients from a multiple linear regression.

CI, confidence interval; JSN, joint space narrowing.

Adjusted by age, body mass index, smoking, alcohol consumption, and education.

*P < 0.05 after Bonferroni correction for multiple comparison

**Table 3:** Odds ratios from a logistic regression model examining the association of grip strength (5 kg) for with individual radiographic features of hand and knee osteoarthritis with a range of less than 10 points.

		Men (*n* = 1,022)		Women (*n* = 1,229)	
		Odds ratio (95% CI)	*P* value	Odds ratio (95% CI)	*P* value
Hand	Subchondral cyst	0.70 (0.62–0.80)	<0.001*	0.81 (0.70–0.95)	0.007
	Subchondral sclerosis	0.99(0.88–1.13)	0.872	1.03 (0.86–1.23)	0.777
	Malalignment	0.86 (0.69–1.07)	0.182	0.79 (0.60–1.03)	0.084
	Erosion	0.82 (0.67–0.99)	0.037	0.90 (0.73–1.11)	0.322
Knee	Tibial attrition	0.95 (0.81–1.11)	0.545	0.96 (0.78–1.17)	0.663
	Femoral sclerosis	0.97 (0.86–1.09)	0.562	0.84 (0.72–0.97)	0.016

CI, confidence interval; JSN, joint space narrowing.

Adjusted by age, body mass index, smoking, alcohol consumption, and education.

*P < 0.05 after Bonferroni correction for multiple comparison

### Associations between grip strength and knee OA

We next evaluated whether knee radiographic OA scores were associated with grip strength using the same type of analysis (Tables 2 and 3). Grip strength showed negative associations with total knee radiographic OA scores in men and women (*p* < 0.001 and *p* = 0.010, respectively), consistent with the hand OA findings. Knee JSN also showed negative associations with grip strength (both *p* < 0.001). The only gender difference concerned a significant negative association between osteophytes and grip strength, which was found only in men (*p* = 0.006). After Bonferroni correction for multiple comparison, grip strength was not associated with tibial attrition or femoral sclerosis of the knee in men or women, and knee osteophyte in men. Overall, grip strength increased as the total radiographic OA score decreased. When the associations between grip strength and knee OA scores were evaluated in a total of 2,415 subjects, the results were not different from those obtained when subjects with hand joint pain were excluded (S3 Table).

## Discussion

In summary, in this analysis of the association between grip strength and hand and knee radiographic OA in a large cohort without hand joint pain, low grip strength was related to hand and knee OA in men and women. For the first time, we found that hand or knee JSN showed the strongest association with low grip strength, regardless of gender. Moreover, the severity of hand osteophytes in women, and subchondral cysts in men was correlated with low grip strength.

In this cross-sectional study, grip strength was associated negatively with hand OA, independent of joint pain. Although similar relationships were found in previous cross-sectional studies [4–8], those studies did not control for hand joint pain, considered to be a mediator of the relationship between grip strength and hand OA, in subjects with hand OA [9]. Individuals with radiographic hand OA have a higher likelihood of hand joint pain compared with those without OA [15]. By excluding subjects with hand joint pain, we eliminated the confounding effect of pain, thereby increasing the certainty of the negative association between grip strength and hand OA. Using this approach, we conclude for the first time that joint pain is not the only factor that leads to weakened grip strength in subjects with hand OA. However, other different results were found in two longitudinal studies: the Oslo hand OA cohort study showed that the amount of hand OA was not associated with grip strength [3], and Chaisson et al. noted in the Framingham longitudinal study that subjects with high maximal grip strength were at increased risk for the development of OA [10]. As a possible explanation for these results, we suspect that most weaker and elderly participants in these studies were lost due to the long follow-up period. The remaining participants, with higher maximal grip strength, were younger at baseline, resulting in more K–L grade results on follow-up X-ray films relative to elderly participants. On the other hand, it is well known that OA mainly occurs in elderly adults, and that the incidence of OA should increase with increasing age. Meanwhile, the mean grip strength decreased with increasing age, from 50 years, and the normative values of mean grip strength for women and men were 22.7 ± 4.4 kg and 36.7 ± 5.5 kg in our study (S1 Table), which was also confirmed by the Korea National Health and Nutrition Examination Survey (KNHANES) [16]. According to the KNHANES results, the grip strength peaked at an age range of 35 to 39 years for both men and women and then decreased after 39 years. All of these results revealed that grip strength was associated negatively with hand OA. Moreover, to clarify further the association between grip strength and hand radiographic OA, we will include relevant measures in our follow-up longitudinal study.

A negative association between knee OA and grip strength was also detected in this study. This relationship has not been considered in other studies, although the relationship between knee muscle strength and knee OA has been analyzed in several previous large cohort studies. A recent systematic review and meta-analysis showed that knee extensor muscle weakness was associated with an increased risk of knee OA development in men and women [17]. Interestingly, the findings of Richard et al. [18] suggested that grip and knee extension strength reflect a common underlying construct, and the relationship between grip strength and knee extension strength showed a good correlation (r = 0.772 to 0.805). Therefore, we can consider hand-grip strength and knee extensor muscle strength as measures of limb-muscle strength. We suspect that low limb-muscle strength is related to the risk of joint OA.

Our results suggest that JSN was the most important type of damage related to low grip strength, and furthermore that the location of OA (hand or knee) did not influence this relationship; our study is the first to demonstrate this negative association between hand JSN and grip strength in such a large cohort. Correspondingly, in a previous study of rheumatoid arthritis, Navarro-Compan et al. indicated that low grip strength was strongly related to hand JSN, although hand JSN in rheumatoid arthritis may be caused partly by the inflammatory state [19]. Regarding hand JSN and low grip strength in osteoarthritic patients, we assume that muscles with reduced strength may fail to play a supportive role in joints. In addition, as one basic previous investigation suggested, a reduced number of muscle cells may affect cartilage gene expression [20], thereby causing JSN. Alternatively, JSN may affect muscle activity, in turn leading to low grip strength. This finding is important for the understanding of the association between grip strength and radiographic OA, and for determination of the etiology of OA.

The connection underlying the negative association between knee JSN and grip strength remains unknown. However, concerning cartilage volumes and muscle strength, the increased cross-sectional area of the thigh muscle was associated with increased patella cartilage and bone volumes, which may benefit knee joint health and reduce the risk of JSN, in a pain-free community-based population [21]. Our hand muscle results are consistent with this suggestion; hand grip strength may be related closely to knee muscle strength. Alternatively, knee JSN may also lead to chronically low levels of physical activity in daily life, which, in turn, may reduce the muscle strength of limbs.[22] We found that the reduced muscle volume associated with JSN was associated not only with the hand, but also with other body joints.

Another result from the present study was that low grip strength was associated with the severity of hand osteophytes in women. Generally, osteophyte formation has been attributed to aging, degeneration, mechanical instability, and disease [23]. Low grip strength is a significant cause of mechanical instability, and usually accompanies aging or degeneration. Thus, a link between low grip strength and osteophytes might be expected. Knoop *et al*. indicated that, regardless of gender, the severity of knee osteophyte formation was associated significantly with low muscle strength in the leg [24]. Our study is the first to indicate a relationship between hand osteophytes and low grip strength, which may be considered to constitute evidence for interactions between muscle tissues and bone metabolism [25].

In addition, hand subchondral cysts were related to low grip strength in men in the present study. Until now, it is widely agreed that the pathogenesis of subchondral cysts remains obscure and uncertain, while Lance L et al. [26] indicated that subchondral cysts alters the mechanical stress of surrounding bone recently, meanwhile, subchondral cysts also accelerate OA, leading to greater pain and disability [27], which cause low muscle strength around the joint.

This study has several limitations. First, the cross-sectional design did not allow us to determine a cause-and-effect relationship between grip strength and radiographic OA, but we intend to undertake further investigations of this question. Second, the Dong-gu study was designed primarily as an investigation of the determinants and prognosis of chronic diseases, and not specifically of grip strength, in middle-aged and aged adults. Thus, we did not have information on all determinants of interest, such as carpal tunnel syndrome and other specific wrist/hand diseases, or on psychosocial factors.

Despite these limitations, to our knowledge, this report is the first to provide some insight into hand grip strength and its relationship to radiographic features of OA in an middle-aged and aged population. A second strength is that we compared radiographic OA data between weight-bearing and non–weight-bearing joints simultaneously, and used a semi-quantitative grading system to evaluate the severity of radiographic OA. Finally, the Dong-gu study enrolled a large number of community-dwelling adults; the original cohort comprised approximately 10,000 subjects, thereby providing a representative sample of the general Korean population. Furthermore, we excluded 164 subjects with hand joint pain, which was not done in similar studies.

In summary, we found that grip strength was associated negatively with hand and knee OA in men and women. Furthermore, lower grip strength was related to the severity of JSN, osteophytes, and subchondral cysts in the hand. Moreover, for the first time, we were able to show that low grip strength had the strongest association with JSN in the same joints, regardless of gender. Finally, our study further underlines the importance of muscle strengthening in the treatment of OA.

## Supporting information

S1 File Supporting information Exel file. Raw data on patients with osteoarthritis in this study.
(XLSX)

S1 Table Distribution of grip strength in adults without hand joint pain by age category. (DOC)

S2 Table Coefficients from a linear regression model examining the association of grip strength (kg) with total and individual radiographic feature scores of hand and knee osteoarthritis in study population (n = 2415) including subjects with hand pain. (DOC)

S3 Table Odds ratios from a logistic regression model examining the association of grip strength (5 kg) for with individual radiographic features of hand and knee osteoarthritis with a range of less than 10 points in study population (n = 2415) including subjects with hand pain. (DOC)
