# Supplementary material for: Association between grip strength and hand and knee radiographic osteoarthritis in Korean adults: Data from the Dong-gu study
Source: PLoS One. 2017 Nov 30;12(11):e0185343. doi: 10.1371/journal.pone.0185343 (PMC5708816; doi:10.1371/journal.pone.0185343)
Supplement: S1 Table — (DOC) [file pone.0185343.s002.doc]

**S1 Table. Distribution of grip strength in adults without hand joint pain by age category.**

| Age group |  | Men |  | Women |
| --- | --- | --- | --- | --- |
|  | N | Mean ± SD | N | Mean ± SD |
| 50-54 | 134 | 40.1 ± 6.2 | 240 | 24.5 ± 4.3 |
| 55-59 | 174 | 38.8 ± 5.6 | 253 | 23.1 ± 4.5 |
| 60-64 | 203 | 36.7 ± 5.5 | 262 | 22.7 ± 4.4 |
| 65-69 | 225 | 34.8 ± 6.1 | 207 | 21.1 ± 4.5 |
| 70-74 | 175 | 33.1 ± 6.1 | 147 | 19.1 ± 3.8 |
| 75-79 | 69 | 30.4 ± 6 | 85 | 18.7 ± 4.2 |
| ≥80 | 42 | 28.9 ± 5.7 | 35 | 16.8 ± 4.6 |
| Total | 1022 | 35.7 ± 6.6 | 1229 | 22 ± 4.8 |
