# Supplementary material for: Association between grip strength and hand and knee radiographic osteoarthritis in Korean adults: Data from the Dong-gu study
Source: PLoS One. 2017 Nov 30;12(11):e0185343. doi: 10.1371/journal.pone.0185343 (PMC5708816; doi:10.1371/journal.pone.0185343)
Supplement: S2 Table — (DOC) [file pone.0185343.s003.doc]

**S2 Table. Coefficients from a linear regression model examining the association of grip strength (kg) with total and individual radiographic feature scores of hand and knee osteoarthritis in study population (n=2415) including subjects with hand pain.**

|  | | Men (*n* = 1,054) | | | | Women (*n* = 1,361) | | | |
| --- | --- | --- | --- | --- | --- | --- | --- | --- | --- |
|  |  | Beta (95% CI) | Standard Beta | Eta | *P* value | Beta (95% CI) | Standard Beta | Eta | *P* value |
| Hand | Total score | -0.153 (-0.205, -0.101) | -0.180 | 0.031 | <0.001* | -0.163 (-0.228, -0.099) | -0.117 | 0.018 | <0.001* |
|  | Osteophyte | -0.022 (-0.043, 0.003) | -0.067 | 0.004 | 0.053 | -0.045 (-0.069, -0.022) | -0.107 | 0.010 | 0.001* |
|  | JSN | -0.091 (-0.119, -0.063) | -0.204 | 0.037 | <0.001* | -0.067 (-0.102, -0.033) | -0.094 | 0.011 | 0.001* |
| Knee | Total score | -0.123 (-0.181, -0.065) | -0.141 | 0.016 | <0.001* | -0.108 (-0.186, -0.029) | -0.068 | 0.005 | 0.007 |
|  | Osteophyte | -0.051 (-0.082, -0.020) | -0.111 | 0.010 | 0.001 | -0.039 (-0.084, 0.005) | -0.045 | 0.002 | 0.084 |
|  | JSN | -0.062 (-0.086, -0.038) | -0.169 | 0.024 | <0.001* | -0.058 (-0.086, -0.031) | -0.106 | 0.012 | <0.001* |

Values are regression coefficients from a multiple linear regression.

CI, confidence interval; JSN, joint space narrowing.

Adjusted by age, body mass index, smoking, alcohol consumption, and education.

*P < 0.05 after Bonferroni correction for multiple comparison
