# Supplementary material for: Association between grip strength and hand and knee radiographic osteoarthritis in Korean adults: Data from the Dong-gu study
Source: PLoS One. 2017 Nov 30;12(11):e0185343. doi: 10.1371/journal.pone.0185343 (PMC5708816; doi:10.1371/journal.pone.0185343)
Supplement: S3 Table — (DOC) [file pone.0185343.s004.doc]

**S3 Table. Odds ratios from a logistic regression model examining the association of grip strength (5 kg) for with individual radiographic features of hand and knee osteoarthritis with a range of less than 10 points in study population (n=2415) including subjects with hand pain.**

|  | | Men (*n* = 1,054) | |  | Women (*n* = 1,361) | |
| --- | --- | --- | --- | --- | --- | --- |
|  |  | Odds ratio (95% CI) | *P* value |  | Odds ratio (95% CI) | *P* value |
| Hand | Subchondral cyst | 0.70 (0.61-0.79) | <0.001* |  | 0.82 (0.71-0.95) | 0.007 |
|  | Subchondral sclerosis | 0.98 (0.86-1.11) | 0.733 |  | 0.96 (0.82-1.14) | 0.652 |
|  | Malalignment | 0.91 (0.73-1.13) | 0.382 |  | 0.76 (0.60-0.97) | 0.024 |
|  | Erosion | 0.82 (0.67-0.99) | 0.038 |  | 0.90 (0.73-1.11) | 0.323 |
| Knee | Tibial attrition | 0.93 (0.80-1.08) | 0.342 |  | 0.96 (0.80-1.16) | 0.669 |
|  | Femoral sclerosis | 0.96 (0.85-1.08) | 0.468 |  | 0.82 (0.72-0.95) | 0.006 |

CI, confidence interval; JSN, joint space narrowing.

Adjusted by age, body mass index, smoking, alcohol consumption, and education.

*P < 0.05 after Bonferroni correction for multiple comparison
